# Modeling of *Escherichia coli* and *Listeria monocytogenes* Inactivation in Human Milk With a Batch UV‐C System: Effect of Agitation, Temperature, and Solids Content

**DOI:** 10.1155/ijfo/4704322

**Published:** 2025-12-10

**Authors:** Maíra Otoni Silva, Eliznara Fernandes Correia, Lahis Hamanda Andrade de Castro, Paulo de Souza Costa Sobrinho

**Affiliations:** ^1^ Department of Nutrition, Federal University of Jequitinhonha and Mucuri Valleys, Diamantina, Minas Gerais, Brazil, ufvjm.edu.br

**Keywords:** bacterial inactivation, batch process, human milk, nonthermal method, UV irradiation

## Abstract

This study was aimed at evaluating the influence of agitation intensity, initial temperature, and total solids content of milk on the inactivation of *Escherichia coli* and *Listeria monocytogenes* by UV‐C radiation, with emphasis on a UV‐C system in batches for human milk. The kinetic parameters of the inactivation of bacteria were estimated by GInaFiT and modeled to obtain a first‐order model. Results showed that the initial temperature of the milk influencing only the *E. coli* inactivation and 4D reductions were achieved with UV‐C dose between 42.7 and 54 J/L for *E. coli* and 29.2 and 48.6 J/L for *L. monocytogenes*, all under agitation conditions above 550 rpm. The UV‐C dose to the first decimal reduction (*δ*) for both bacteria was approximately fourfold greater at 220 rpm than at 880 rpm. There is a statistically significant interaction between the effects of agitation intensity and total solids content for the prediction models of the *p* parameter of both bacteria. The intensity of agitation overcame the short penetration depth of UV radiation by exposing bacteria to irradiated areas more frequently in the inactivation of pathogenic bacteria in milk, and this batch UV‐C system demonstrated potential for use in human milk processing.

## 1. Introduction

Human milk is more than a suitable food for the newborn′s nutrition; it is also considered a medical product of human origin [1]. The benefits of human milk are so significant and diverse that it is the gold standard for low birth weight infant feeding [2]. Consolidation of the benefits of human milk has increased worldwide interest in creating and maintaining human milk banks to meet the need for donated human milk [2–4].

Several countries, including Brazil, have developed their guidelines to implement and regulate human milk banks, and Holder pasteurization (62.5°C for 30 min) is the most adopted treatment as a criterion for the safety of donated milk [4–6]. However, studies indicate that several protective factors in human milk are wholly or partially destroyed by heat treatment, thus reducing its biological value compared to raw human milk [7–9].

In recent years, several nonthermal methods have been proposed for processing human milk to minimize the loss of nutritional or biological components when compared to Holder pasteurization, especially physical methods using high pressure [10–13] and ultraviolet (UV‐C) [14–18].

Ultraviolet radiation cannot penetrate food, and due to this limitation, it is traditionally used to disinfect surfaces and treat drinking water. However, several studies have circumvented the high absorption coefficients of liquid foods, such as egg [19], fruit juices [20–23], soy milk [24], cow milk [25–29], and human milk [14, 15, 30–33], and successfully promoted microbial reduction without affecting sensory characteristics compared to thermal pasteurization.

One of the ways to bypass liquids with a high absorption coefficient, such as human milk, is to promote a flow of the fluid around the source of ultraviolet radiation [14, 22, 29, 34, 35]. Christen et al. [14] demonstrated the potential of UV‐C irradiation as an alternative method to thermal pasteurization of human milk by applying a low‐velocity agitation (500 rpm). As a result, they observed a high positive correlation between the solids content of human milk and the applied dose of UV‐C. However, they did not evaluate the milk agitation influence on the bacterial inactivation. Additionally, they did not estimate an inactivation model different from that of the linear model. Other inactivation studies that used UV‐C radiation also kept the stirring speed of the liquid constant [31, 33].

In addition to agitation intensity, the temperature of the liquid can influence the inactivation of bacteria by UV‐C irradiation, especially mild heat treatments [23, 24], as well as temperatures below 20°C [24]. Human milk banks establish strict temperature control under the cold chain so that the temperature should not exceed 5°C, except in Holder pasteurization [6].

This study was aimed at evaluating the influence of agitation intensity, initial temperature, and total solids content of milk on the inactivation of *Escherichia coli* and *Listeria monocytogenes* by UV‐C, with emphasis on a UV‐C system in batches for small volumes of milk, according to the reality of human milk banks. *E. coli* was studied because it is a bacterium in the coliform group, which serves as a criterion for quality control in human milk banks*. L. monocytogenes* was chosen due to recent cases of its detection in human milk samples [36, 37].

## 2. Material and Methods

### 2.1. Milk Samples

Due to the limited availability of human milk for all the experiments, UHT milk, whole and skimmed cow milk, was used to develop the predictive model with subsequent validation using human milk. Five healthy lactating women in home facilities donated the human milk samples used in the study. All donors gave written consent for their donations to be used in research, and the Human Research Ethics Committee approved the study protocol (Number 3.699.107). Each donor received a kit containing a 200 mL sterilized glass bottle for storing expressed milk. Before the donation, the volunteers received instructions, in a folder and video, on the procedures for collecting breast milk recommended by the Brazilian network of human milk banks. All breast milk samples were received from donors frozen in a glass bottle, transported to the laboratory, and stored at −80°C until carrying out the experiments.

### 2.2. Analysis of Milk Samples

The pH of the milk sample, cow or human, was determined using a calibrated pH meter (Akso, Brazil), and the acidity was determined following the AOAC 947.05 method [38] using a volume of 10 mL of milk for human milk samples. The total solids (percentage) content of milk was determined by gravimetry following AOAC method 990.20 [38], and fat content (percentage) was determined by AOAC method 989.05 [38].

### 2.3. Bacterial Inoculum Preparation

*E. coli* (ATCC 25922) and *L. monocytogenes* (CLIST 03436, CLIST 2032, CLIST 2035, CLIST 2043, and CLIST 2048) were used in this study. *Listeria* strains were donated by the Oswaldo Cruz Foundation (Brazil). The strain was grown in Brain Heart Infusion (BHI) broth for 24 h at 35°C. The inoculum of *L. monocytogenes* strains was prepared by mixing and homogenizing 1 mL aliquots of each strain grown individually in BHI broth, and then this mixture was used to prepare a bacterial suspension in buffered peptone water adjusted to 0.5 on the McFarland scale. This prepared suspension was used to inoculate a population of approximately 10^6^ CFU/mL of milk sample.

### 2.4. Bacterial Analysis

Aliquots of irradiated and nonirradiated milk samples were diluted with saline peptone water, and bacteria were enumerated by the spread plate method on plate count agar (Himedia, India). Plates were incubated for 24–48 h at 35°C. Additionally, in the validation experiments, in addition to plate count agar, plates of Eosin Methylene Blue Agar (Oxoid, United Kingdom) and Listeria Chromogenic Agar (ALOA) (Harlequin Listeria Chromogenic Agar, Neogen, United States) were used for *E. coli* and *L. monocytogenes*, respectively, incubated for 24–48 h at 35°C.

Additionally, prior to the experiments, the UHT milk sample was evaluated for the absence of viable mesophilic bacteria by inoculating 0.1 mL onto plate count agar, and human milk samples were analyzed for the absence of *E. coli* and *L. monocytogenes* using Eosin Methylene Blue Agar (Oxoid, United Kingdom) and Listeria Chromogenic Agar (ALOA) (Harlequin Listeria Chromogenic Agar, Neogen, United States), respectively.

### 2.5. UV‐C Treatment

**Figure 1:**
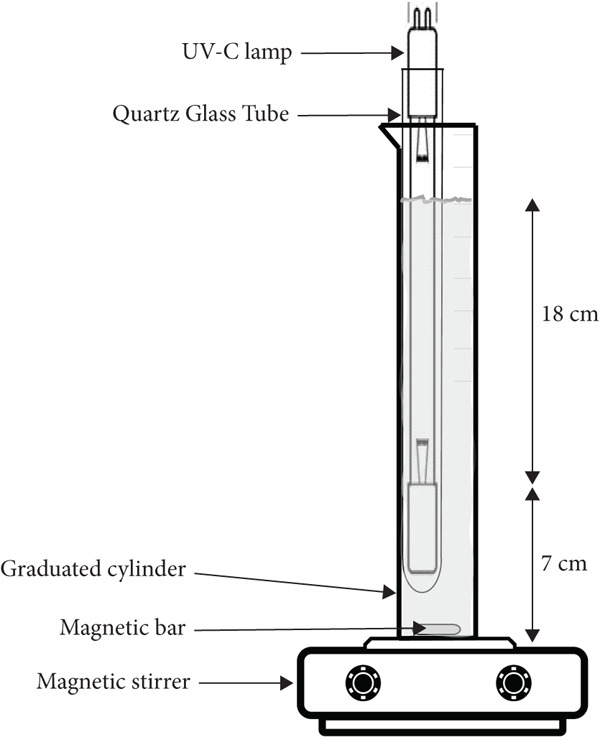
Diagram of the assembled UV‐C device for processing milk.

Each milk sample (190 mL), cow or human, was transferred to a 250‐mL borosilicate graduated cylinder (0.046 m diameter and 0.305 m height). The definition of the volume to be processed by batch was based on the capacity of the bottle used by the Brazilian milk bank network, which is 0.2 L. The ultraviolet light (UV‐C) device used (Figure 1) consists of a lamp (TUV 16W 4P SE UNP/32, Philips) placed inside a quartz tube (diameter of 0.025 m and height of 0.315 m) and is arranged vertically in contact with the test tube wall.

During UV‐C experimental treatment procedure, agitation of milk sample was generated by magnetic stirring using a magnetic bar (0.005 × 0.02 m). Samples were exposed to UV‐C radiation for 20 min, with interruptions of 40–50 s at 4‐min intervals to collect 1 mL aliquots to quantify the surviving population. The UV‐C treatment was realized in an environment with a temperature of 20^°^C ± 2^°^C. The dose (joules per liter) was estimated multiplying the exposure time by UV‐C irradiance of the lamp. UV‐C irradiance (45 mW/L) was determined with the configurations adopted (Figure 1) by chemical actinometry (Rahn, 1997). The actinometric solution, prepared with 0.6 M iodide and 0.1 M iodate in a 0.01 M borate buffer (pH 9.25), was exposed without stirring for 60 s using the apparatus set up for the experiment as shown in Figure 1. Irradiance was determined under static conditions as an average dose in 190 mL of the actinometric solution based on three replicates.

### 2.6. Experimental Design, Data Modeling, and Statistical Analysis

To evaluate the influence of agitation intensity, initial temperature, and total solids content of milk on the inactivation kinetic parameters of *E. coli* and *L. monocytogenes* during UV‐C treatment, a full factorial design (2^3^) was performed with three replicates at the central point, which totaled 11 experiments. The center point was 12.5°C, 550 rpm, and 10.95% of total solids. The agitation was evaluated at 220 and 880 rpm, and the milk temperature was evaluated at 5°C and 20°C, while the solids content was evaluated in skimmed cow milk (9.2%), whole cow milk (12.7%), and human milk (11.0%) used in validation. The temperature of 20°C was due to the inadequate practice of keeping donated human milk at room temperature until it was administered to the baby. The experimental central point and validation were estimated from three experiments conducted on independently grown cultures.

The difference between surviving bacteria and initial counts (log_10_ N/N_0_) was plotted as a UV‐C dose (joules per liter) function. The inactivation kinetic parameters of bacteria in UV‐C‐treated milk were determined with GInaFiT (Version 1.8) for Microsoft Excel [39]. The goodness‐of‐fit of the model was evaluated by root mean square error (RMSE), coefficient of determination (*R* ^2^ adjusted), and bias and accuracy factors calculated according to [40] (supporting information [available here]). The models evaluated were Weibull [41], log‐linear [42], log‐linear shoulder [43], and log‐linear tail [43] (supporting information).

The kinetic parameters were modeled through the standard least squares model to obtain a first‐order model with interaction, considering lack of fit, model significance, *R* ^2^ adjusted, and normality of the residuals. The significant factors of the model were determined considering the significance level of 0.05.

## 3. Results and Discussion

### 3.1. Characterization of Milk Samples

The results of the compositional and physicochemical analyses of the milk are presented in Table 1. The composition of human milk donated in this study is generally consistent with that reported in other studies [14, 44, 45]. The acidity and pH values indicate that milk samples are low‐acid foods and susceptible to bacterial proliferation, with cow milk showing less variability in parameters than human milk, which is explained by standardization during processing in the dairy industry. The results show that human and whole cow milk had similar solids contents, but the average fat content was higher in human milk samples.

**Table 1:** Physicochemical and composition characteristics of the milk samples used in UV‐C irradiation.

**Parameter**	**Milk sample**		
	**Whole cow milk**	**Skimmed cow milk**	**Whole human milk**
pH	6.5 (0.2)^a^	6.4 (0.5)	6.5 (0.2)
Acidity (°Dornic)	17.5 (0.5)	19.5 (0.7)	11.3 (2.8)
Total solids (%)	12.7 (0.3)	9.3 (0.3)	12.4 (0.5)
Fat content (%)	3.5 (0.1)	< 0.1	3.9 (0.3)

^a^Values represent the mean (standard deviation) of the three independent lots, except for human milk, which is from five donors.

### 3.2. Bacterial Inactivation in Milk Samples During UV‐C Treatment

The kinetic parameters of the inactivation of *E. coli* and *L. monocytogenes* during UV‐C treatments are shown in Tables 2 and 3, respectively. The results confirm the versatility of the Weibull models [41, 46] for predicting bacterial inactivation using UV irradiation, which, in general, in this study obtained better adjustments than the log‐linear models [42] or log‐linear with shoulder or tail [43]. However, all three models accurately fitted the bacterial inactivation data in cow and human milk, with *R* ^2^ adjusted values ranging from 0.92 to 0.99 and RMSE values ranging from 0.43 to 0.02.

**Table 2:** Kinetic parameters of *E. coli* inactivation in milk treated by UV‐C irradiation under different conditions of agitation, solids, and temperature.

**Run**	**Revolutions per minute (rpm)**	**Total solids (%)**	**Temperature (°C)**	**Weibull model [** **41** **]**						**Log-linear with shoulder [** **43** **] or log-linear model [** **42** **]**			
				**δ**	**p**	**4D**	$R_{adj}^{2}$	**RMSE**	**Bias/accuracy**	**k**_max_	**SI**	$R_{adj}^{2}$	**RMSE**
1	220	9.2	5 (5–20)^a^	39.47 (3.25)	2.28 (0.5)		0.96	0.16	1.00/1.03	0.15 (0.03)	24.62 (5.97)	0.94	0.20
2	220	9.2	20 (20–32)	33.31 (1.41)	2.20 (0.18)		0.99	0.08	1.00/1.01	0.20 (0.02)	22.12 (3.27)	0.98	0.16
3	220	12.7	5 (5–25)	37.92 (4.20)	2.12 (0.56)		0.94	0.20	1.00/1.02	0.16 (0.03)	25.15 (5.16)	0.95	0.19
4	220	12.7	20 (20–32)	38.33 (1.13)	1.2 (0.07)		0.99	0.03	1.00/1.00	0.06 (0.00)	—	0.99	0.05
5	880	9.2	5 (5–28)	7.39 (1.25)	0.79 (0.06)	42.7	0.99	0.14	1.00/1.03	0.20 (0.01)	—	0.98	0.24
6	880	9.2	20 (20–32)	1.93 (0.66)	0.43 (0.04)	47.5	0.99	0.13	0.99/1.05	0.29 (0.07)^b^	—	0.92	0.43
7	880	12.7	5 (5–25)	15.41 (1.40)	1.04 (0.07)		0.99	0.08	1.00/1.01	0.16 (0.00)	—	0.99	0.08
8	880	12.7	20 (20–32)	8.75 (0.92)	0.82 (0.04)	47.5	0.99	0.09	1.00/1.02	0.19 (0.01)	—	0.99	0.17
9	550	10.95	12.5 (12–30)	14.15 (2.05)	1.04 (0.10)	54.0	0.99	0.14	1.00/1.03	0.17 (0.01)	—	0.99	0.13
10	550	10.95	12.5 (12–30)	13.40 (2.70)	1.07 (0.14)	49.2	0.98	0.21	1.00/1.04	0.19 (0.01)	—	0.98	0.19
11	550	10.95	12.5 (12–30)	17.66 (4.23)	1.26 (0.25)	53.5	0.97	0.28	1.00/1.04	0.18 (0.01)	—	0.97	0.28
Validation with human milk													
Observed													
	880	11.0	7 (7–29)	5.25 [1.42; 9.09]^c^	0.72 [0.51; 0.93]^c^	36.7	0.99	0.18	0.99/1.04	0.25 (0.03)^b^	—	0.97	0.31
Predicted				5.15 [−1.56; 11.87]	0.53 [0.24; 0.83]								

^a^Initial and final milk temperatures.

^b^Geeraerd tail model.

^c^95% confidence interval.

**Table 3:** Kinetic parameters of *L. monocytogenes* inactivation in milk treated by UV‐C irradiation under different conditions of agitation, solids, and temperature.

**Run**	**Revolutions per minute (rpm)**	**Total solids (%)**	**Temperature (°C)**	**Weibull model [** **41** **]**						**Log-linear with shoulder [** **43** **] or log-linear model [** **42** **]**			
				**δ**	**p**	**4D**	$R_{adj}^{2}$	**RMSE**	**Bias/accuracy**	**k**_max_	**SI**	$R_{adj}^{2}$	**RMSE**
1	220	9.2	5 (5–28)^a^	30.21 (1.75)	1.80 (0.16)		0.99	0.09	1.00/1.01	0.17 (0.01)	17.66 (2.66)	0.99	0.11
2	220	9.2	20 (20–32)	25.16 (2.02)	1.58 (0.15)		0.99	0.11	1.00/1.01	0.19 (0.02)	13.97 (3.56)	0.98	0.16
3	220	12.7	5 (5–25)	28.98 (2.96)	0.96 (0.12)		0.98	0.08	1.00/1.01	0.08 (0.01)	—	0.99	0.07
4	220	12.7	20 (21–31)	27.65 (4.96)	1.09 (0.23)		0.96	0.16	1.00/1.02	0.09 (0.01)	—	0.97	0.14
5	880	9.2	5 (5–28)	2.13 (1.10)	0.53 (0.08)	29.2	0.98	0.29	1.00/1.02	0.34 (0.03)	—	0.98	0.27
6	880	9.2	20 (20–32)	4.03 (0.50)^b^	0.71 (0.05)	30.2	0.99	0.07	1.00/1.01	0.35 (0.03)	—	0.98	0.21
7	880	12.7	5 (5–27)	16.22 (0.47)	1.27 (0.03)	48.6	0.99	0.04	1.00/1.02	0.22 (0.00)	7.18 (0.91)	0.99	0.05
8	880	12.7	20 (20–31)	6.59 (2.01)	0.81 (0.11)	36.7	0.98	0.27	0.98/1.09	0.26 (0.02)	—	0.98	0.25
9	550	10.95	12.5 (12.5–30)	13.13 (1.69)	1.10 (0.09)	47.0	0.99	0.14	1.00/1.03	0.20 (0.01)	—	0.99	0.15
10	550	10.95	12.5 (12.6–30)	10.37 (0.92)	0.95 (0.05)	44.8	0.99	0.09	1.00/1.03	0.20 (0.00)	—	0.99	0.09
11	550	10.95	12.5 (12.5–29)	15.37 (1.03)	1.29 (0.06)	45.4	0.99	0.09	1.00/1.02	0.22 (0.01)	—	0.98	0.25
Validation with human milk													
Observed													
	880	11.0	7 (7–29)	7.67 [3.51; 11.79]^c^	0.85 [0.63; 1.06]^c^	40.0	0.99	0.15	0.99/1.06	0.22 (0.01)	—	0.98	0.20
Predicted				7.52 [0.23; 15.3]	0.89 [0.54; 1.24]								

^a^Initial and final milk temperatures.

^b^Weibull tail model.

^c^95% confidence interval.

**Figure 2:**
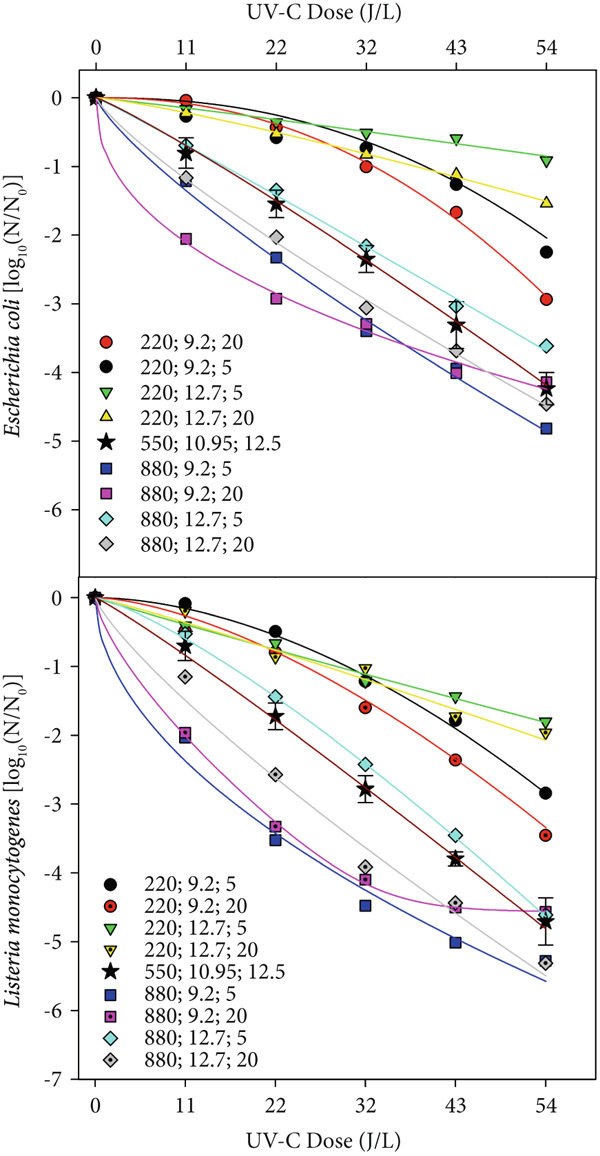
Survival curves of the bacteria in milk treated by UV‐C irradiation fitted by the Weibull model under different conditions of agitation (220, 550, and 880 rpm), solids (9.2%, 10.95%, and 12.7%), and milk temperature (5°C, 12.5°C, and 20°C).

The kinetic profile was similar between the two bacteria (Figure 2) under the analyzed conditions, and *E. coli* proved to be, on average, 1.15 times more resistant to the first decimal reduction (*δ*) than *L. monocytogenes* and with an inactivation rate maximum (*k*_ma*x*_) of 0.29, while *L. monocytogenes* was 0.35. This similarity was also observed by Christen et al. [14], but between *E. coli* and *Staphylococcus aureus*. The greater resistance to UV‐C inactivation of *E. coli*, compared to *L. monocytogenes*, was also observed in apple juice [47] and contrasts with the assertion that gram‐positive bacteria are more resistant to UV‐C than gram‐negative bacteria [48].

The initial resistance to inactivation (shoulder effect) was more pronounced for *E. coli* and only under agitation conditions of 220 rpm. Delta values ranged from 1.93 to 39.47 for *E. coli* and 2.13 to 30.21 for *Listeria*. Regarding the *p* parameter of the Weibull model, the different experimental conditions promoted very different behaviors for the bacteria, with *p* values ranging from 0.72 (convex) to 2.28 (concave) for *E. coli* and 0.53 to 1.80 for *Listeria*. 4D reductions were achieved with UV‐C dose between 42.7 and 54 J/L for *E. coli* and 29.2 and 48.6 J/L for *L. monocytogenes*, all under agitation conditions above 550 rpm.

The thermal D value for *L. monocytogenes* and *E. coli* STEC in whole cow′s milk at 62.8°C is 33.8 and 16.9 s, respectively [49]. Thus, the 4D time is approximately 135 s (2.25 min) and 68 s (1.12 min) for *L. monocytogenes* and *E. coli*, respectively. These values (4D) are much lower than those obtained in this work for UV‐treated whole milk, which ranged from 36 to 48 min. However, human milk has a diversity of bioactive compounds whose preservation is desirable, such as immunoglobulins and leptin [7]. From this perspective, Holder pasteurization causes a significant loss in the concentration of bioactive components present in human milk, an effect that is not significant with UV processing [50].

Christen et al. [17] found that the retention rate of immunoglobulin A, lactoferrin, and lysozyme with UV‐C irradiation was above 75%, while the highest retention rate with Holder pasteurization was 49%. Furthermore, the study examined the bacterial growth rate in human milk treated with UV and Holder pasteurization and observed that the rate in UV‐treated milk was not different from that in untreated milk, but in milk treated with thermal pasteurization, the growth rate was doubled compared to untreated human milk. Two other positive factors for UV‐C milk treatment were the potential to reduce aflatoxin M1 [51] and to increase vitamin D3 concentration [52].

### 3.3. Modeling Kinetic Parameters

Based on the kinetic parameters presented in Tables 2 and 3, the predictive models for the parameters of the Weibull model were adjusted for the independent variables (Table 4 and Figure 3), with the initial temperature of the milk influencing only the *E. coli* inactivation. The recognized psychrotrophic behavior of *Listeria* monocytogenes may explain the lack of influence of milk temperature on its inactivation, unlike *E. coli*, a mesophilic bacterium. The temperature (between 4°C and 18°C) influenced the inactivation by UV‐C of another mesophilic bacterium (*Salmonella*) in soy milk [24]. Milk temperature during UV‐C treatment was not controlled, so the temperature of the milk during irradiation gradually increased, which may have contributed to the weak influence of the initial milk temperature on the inactivation parameters. The initial milk temperature significantly influenced only the inactivation of *E. coli* in the interaction effect with agitation (Table 4). However, the observed maximum variation of 23°C, with a maximum temperature of 32°C, indicates that this rise in milk temperature during treatment must not have contributed significantly to the thermal inactivation of the evaluated bacteria.

**Table 4:** Parameter estimates of the secondary model.

**Factors**	***Escherichia coli***		***Listeria monocytogenes***	
	**δ**	**p**	**δ**	**p**
Intercept	20.70^a^	1.29	16.35	1.10
Agitation (A)	−14.44	−0.59	−10.38	−0.26
Total solids (TS)		−0.197		
Temperature (T)				
A∗TS				0.27
A∗T		0.225		
TS∗T				
*p* value	0.0008	0.0007	0.0006	0.0009
Lack of fit	*p* = 0.13	*p* = 0.17	*p* = 0.11	*p* = 0.66
*R* ^2^ adjusted	0.90	0.90	0.85	0.82

^a^Statistically significant model parameters (*p* < 0.05).

Figure 3Interaction plot of the kinetic parameters of the Weibull model of *E. coli* and *L. monocytogenes* inactivation in milk by UV‐C treatment as a function of agitation, (a) total solids, and (b) initial temperature of the milk.

**Figure:**
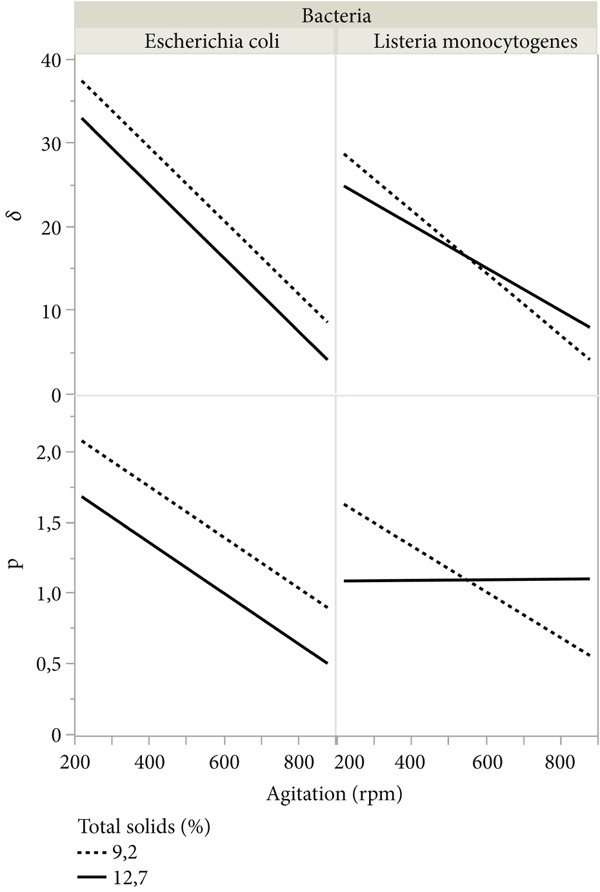
(a)

**Figure:**
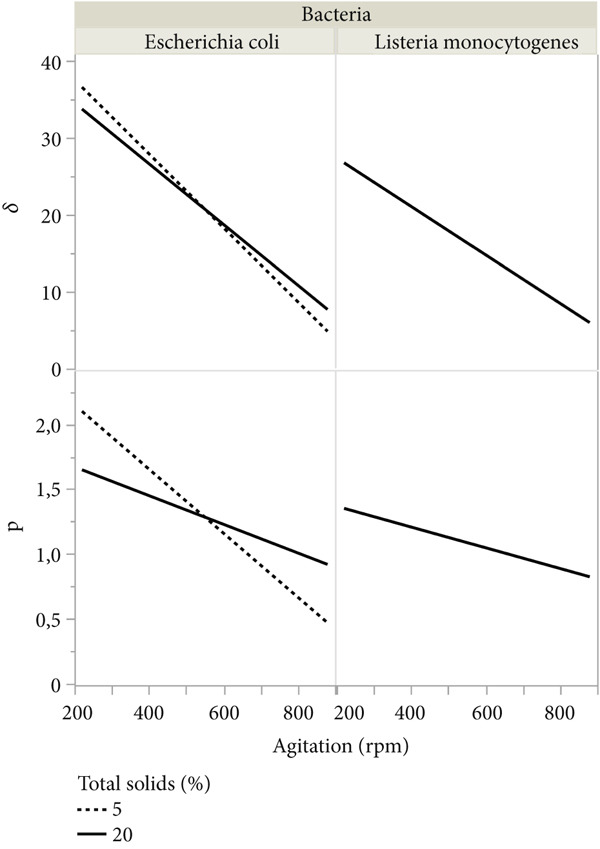
(b)

The UV‐C dose to the first decimal reduction (*δ*) for both bacteria was approximately fourfold greater at 220 rpm than at 880 rpm, showing that agitation intensity at 880 rpm quadrupled the dose effect of UV‐C and the importance of agitation to circumvent the high absorption coefficients of milk. In this study, the agitation was the variable that had the most significant influence on the inactivation parameters (Figure 3), adjusting different models of the behavior, from descending (tail) to ascending (shoulder), passing through the log‐linear (Figure 2). Agitation above 500 rpm promotes a more homogeneous mixture of bacteria around the lamp to eliminate the shoulder effect observed in agitation at low rotations (220 rpm). Linearity in UV‐C inactivation (*p* close at 1) was observed at agitation conditions of approximately 550–600 rpm. The fitted model predicts behavior with downward concavity (*p* > 1) at agitations slower than 550–600 rpm. Interestingly, the adjusted model for *L. monocytogenes* predicts convergence in the *p* parameter value (1.12) with the increase in the total solids content, regardless of the agitation condition, showing that inactivation of *Listeria* is not influenced by agitation of milk with the highest percentage of total solids. For both bacteria, total solids content significantly influenced only the parameter p of the Weibull model (Figure 3).

The results show a statistically significant interaction between the effects of agitation intensity and total solids content for the prediction models of the *p* parameter of both bacteria, showing that the interaction influences the concavity of the model curve. The interaction plot (Table 4 and Figure 3) of the kinetic parameters indicates that the UV‐C inactivation profile of bacteria in milk depends on the agitation intensity, solids content and, for *E. coli*, to a lesser extent, the milk temperature.

The inactivation model was validated using human milk experiments for both bacteria. As seen in Tables 2 and 3, the evaluation parameters obtained a goodness fit in the secondary model, both for *E. coli* with $R_{\text{adj}}^{2}$, bias, accuracy, and RMSE of 0.99, 0.99, 1.04, and 0.17, respectively, and for *L. monocytogenes* with $R_{\text{adj}}^{2}$, bias, accuracy, and RMSE of 0.99, 0.99, 1.06, and 0.15, respectively. The kinetic parameters observed for human milk in the validation condition overlap with the 95% confidence interval predicted by the secondary model. Therefore, this model can be used to predict the inactivation of *E. coli* and *L. monocytogenes* in milk, including human milk, treated with UV‐C.

The interaction between agitation speed and solids content is well known in UV inactivation. However, several studies did not evaluate the influence of agitation speed on the UV inactivation of pathogenic bacteria, including human milk [14, 31], soymilk [24], skim milk [53], egg [19], and water [54]. I would like to point out that in some of these studies, there is no description of the agitation speed used. Furthermore, we did not identify any studies that evaluated these variables simultaneously in a factorial design for practical optimization. Regarding non‐log‐linear fits, Christen et al.′s study [14], for example, the number of samples was insufficient to determine whether non‐log‐linear models would be more appropriate than a linear model.

The study confirms the versatility of the Weibull model to describe the inactivation of bacteria by UV‐C irradiation in liquid foods. The intensity of agitation overcame the short penetration depth of UV radiation by exposing bacteria to irradiated areas more frequently in the inactivation of pathogenic bacteria in milk, and this batch UV‐C system demonstrated the potential to be used in processing human milk. According to our findings, the influence of the temperature of the liquid/food on UV inactivation is dependent on the bacteria, as it significantly influenced the inactivation of *E. coli* (gram‐negative) but not the inactivation of *L. monocytogenes* (gram‐positive). This study contributed to a better understanding of the factors that influence the inactivation of pathogenic bacteria in liquid foods by processing nonthermal ultraviolet radiation technology.

## Conflicts of Interest

The authors declare no conflicts of interest.

## Author Contributions

**Paulo de Souza Costa Sobrinho:** conceptualization, methodology, investigation, visualization, writing – original draft, funding acquisition. **Eliznara Fernandes Correia:** methodology, investigation, supervision. **Lahis Hamanda Andrade de Castro:** investigation. **Maíra Otoni Silva:** methodology, investigation, visualization, writing – original draft.

## Funding

No funding was received for this manuscript.

## Supporting information


**Supporting Information** Additional supporting information can be found online in the Supporting Information section. The supporting information describes the mathematical models of bacterial inactivation presented in the manuscript, as well as the equations for the model adjustment criteria (accuracy and bias factors).

## Data Availability

The data that support the findings of this study are available from the corresponding author upon reasonable request.
