# Supplementary material for: Modeling of Escherichia coli and Listeria monocytogenes Inactivation in Human Milk With a Batch UV‐C System: Effect of Agitation, Temperature, and Solids Content
Source: Int J Food Sci. 2025 Dec 10;2025:4704322. doi: 10.1155/ijfo/4704322 (PMC12696034; doi:10.1155/ijfo/4704322)
Supplement: Supplementary file 1 — Supporting Information Additional supporting information can be found online in the Supporting Information section. The supporting information describes the mathematical models of bacterial inactivation presented in the manuscript, as well as the equations for the model adjustment criteria (accuracy and bias factors). [file IJFO-2025-4704322-s001.docx]

Weibull model (Mafart et al., 2002)

$$log\left( \frac{N}{N_{0}} \right)=-\left( \frac{D}{\delta} \right)^{p}$$

Log-linear model (Bigelow & Esty, 1920)

$$log\left( \frac{N}{N_{0}} \right)=-{k_{max}\times D}/{ln(10)}$$

Log-linear shoulder model (Geeraerd et al., 2000)

$$log\left( \frac{N}{N_{0}} \right)=-{k_{max}\times D}/{ln(10)}+log\left( {e^{k_{max}\times SI}}/\left( 1+\left( e^{k_{max}\times SI}-1 \right)\times e^{-k_{max}\times D} \right) \right)$$

Log-linear tail model (Geeraerd et al., 2000)

$$logN=log\left( {10}^{logN_{0}}-{10}^{logN_{res}} \right)\times e^{{-k}_{max}\times D}+{10}^{logN_{res}}$$

Where N is the surviving population after UV-C dose (log CFU/mL); k_max_ = maximum inactivation rate; N_0_ = is the population before UV-C treatment (log CFU/mL); N_res_ = residual subpopulation (log CFU/mL); SI = duration of shoulder effect; δ = time to first log-reduction of first subpopulation; p = shape of inactivation curve and D = dose of ultraviolet radiation

Accuracy and Bias factors (*A_f_* and *B_f_*) were calculated according to Equations (1) and (2), respectively (Ross, 1996).

$A_{f}={10}^{\frac{\sum\left| \log\left( \frac{{\log\left( CFU/mL \right)}_{predicted}}{{\log\left( CFU/mL \right)}_{observed}} \right) \right|}{n}}$ (1)

$B_{f}={10}^{\frac{\sum\log\left( \frac{{\log\left( CFU/mL \right)}_{predicted}}{{\log\left( CFU/mL \right)}_{observed}} \right)}{n}}$ (2)
